# 3D Cell Migration Studies for Chemotaxis on Microfluidic-Based Chips: A Comparison between Cardiac and Dermal Fibroblasts

**DOI:** 10.3390/bioengineering5020045

**Published:** 2018-06-12

**Authors:** Sandra Pérez-Rodríguez, Esther Tomás-González, José Manuel García-Aznar

**Affiliations:** 1 Multiscale in Mechanical and Biological Engineering, Department of Mechanical Engineering, University of Zaragoza, 50018 Zaragoza, Spain; sandrapr@unizar.es (S.P.-R.); estogon@unizar.es (E.T.-G.); 2 Aragon Institute of Engineering Research, University of Zaragoza, 50018 Zaragoza, Spain

**Keywords:** fibroblast migration, 3D collagen scaffold, chemotaxis, microfluidics

## Abstract

Fibroblast migration to damaged zones in different tissues is crucial to regenerate and recuperate their functional activity. However, fibroblast migration patterns have hardly been studied in disease terms. Here, we study this fundamental process in dermal and cardiac fibroblasts by means of microfluidic-based experiments, which simulate a three-dimensional matrix in which fibroblasts are found in physiological conditions. Cardiac fibroblasts show a higher mean and effective speed, as well as greater contractile force, in comparison to dermal fibroblasts. In addition, we generate chemical gradients to study fibroblast response to platelet derived growth factor (PDGF) and transforming growth factor beta (TGF-β) gradients. Dermal fibroblasts were attracted to PDGF, whereas cardiac fibroblasts are not. Notwithstanding, cardiac fibroblasts increased their mean and effective velocity in the presence of TGF-β. Therefore, given that we observe that the application of these growth factors does not modify fibroblasts’ morphology, these alterations in the migration patterns may be due to an intracellular regulation.

## 1. Introduction

Fibroblasts are the most abundant cells in connective tissue and are responsible for secreting extracellular matrix, which is rich in collagen, to give tissue consistency. After tissue damage, fibroblasts from nearby healthy areas proliferate and are able to respond to molecular signals and migrate to the damaged area [1]. There, some of them are transformed into myofibroblasts, a specialized form of fibroblast capable of synthesizing lost connective tissue, whereas the others maintain their original state [2,3,4]. Once damage is repaired, myofibroblasts die by apoptosis [4]. Some of these signalling molecules involved in wound healing, both in epithelium and in cardiac tissue, are platelet derived growth factor (PDGF) and transforming growth factor beta (TGF-β) [5].

The PDGF family is a multifunctional group of proteins involved in a multitude of physiological functions, among which their necessary implication for the division of fibroblasts, especially myofibroblasts, and their contribution to the maintenance of connective tissue stands out. Thus, PDGF is considered as an essential factor for wound healing. In addition, expression of this factor has been shown to increase in cardiac tissue after a heart attack, regulating inflammation, angiogenesis, and fibrosis to repair caused damage [6]. TGF-β belongs to a family with three important isoforms, all of which are present in wound healing, which are secreted as inactive precursors that must be activated by proteolytic cutting and dimerization, allowing it to be attached to its receptors. This union activates pathways aimed at blocking the cell cycle or activating an alternative differentiation program [7]. The presence of both factors has been studied in epidermal wounds, determining that both have positive effects on healing [5]. PDGF is a chemotherapeutic agent of epithelial fibroblasts, which favours their migration to the damaged area, as well as stimulating their proliferation. On the other hand, TGF-β has functions of formation and remodelling of the extracellular matrix, since it is capable of modifying the metabolism of fibroblasts [5,8]. This effect has also been studied in cardiac fibroblasts, determining that TGF-β increases the expression of profibrotic genes and proteins present in the extracellular matrix, as well as the secretion of collagen I [9]. Other studies have demonstrated the transdifferentiation capacity of TGF-β, transforming lung fibroblasts into myofibroblasts [10]. However, the migration patterns of dermal and cardiac fibroblasts because of these factors have not often been studied.

In vivo, fibroblast migration occurs in three dimensions (3D), so it is more appropriate to work with a three-dimensional platform that simulates the physiological environment. In addition, literature has showed that 3D migration patterns differ from those in 2D. For example, Wu et al., determined that human fibrosarcoma cells showed an anisotropic migration in 3D, but not in 2D [11]. One alternative to develop this 3D cell culture is by means of microfluidics devices. Microfluidics consists of engineering technology designed to manipulate fluids at micrometric scales [12], allowing the miniaturization, integration, and automation of different processes [13]. Thus, these devices allow recreation of the physiological cellular environment in vitro [13,14,15,16,17,18] and, in particular, they are especially adequate for the application of chemical gradients [15]. In this study, we used microfluidic devices to simulate PDGF and TGF-β gradients, and analyse the 3D migration patterns of dermal and cardiac fibroblasts under these gradients.

## 2. Materials and Methods 

### 2.1. Cell Culture

We used two commercial human primary lines of fibroblasts. One was Normal Human Dermo Fibroblasts (NHDF) that came from the dermis of adult skin and were grown in insulin-supplemented Fibroblast Basal Medium, Fibroblast Growth Factor basic human recombinant (rhFGF-B), Gentaminicin sulfate/Amphotericin (GA-1000), and 2% of Fetal Bovine Serum (FBS) (medium FGM-2, Lonza, Basel, Switzerland). The other was Normal Human Cardiac Fibroblasts (NHCF-v), which were isolated from healthy adult heart ventricles and cultured in insulin-supplemented FBM media, rhFGF, GA-1000, and 10% of FBS (medium FGM-3, Lonza, Basel, Switzerland). Passages used were from 5 to 8 in NHDF and from 2 to 5 in NHCF-v. 

### 2.2. Fabrication of Microfluidic Devices

Microfluidic devices were manufactured following the Shin et al. (2012) protocol [17], explained below. The polydimethylsiloxane (PDMS) mould consisted of a degassed mixture of the base and curing agent of Sylgard 184 silicone elastomer in a 10:1 ratio, which was poured onto the wafer with the desired design. After incubation at 80 °C overnight, the mould was separated and perforated, according to the designed pattern, and autoclaved. The moulds were then plasma treated and sealed on 35 mm diameter glass bottom plates (µ-Dish 35 mm, ibidi, Planegg/Martinsried, Germany). Finally, devices were filled with a treatment of Poly-d-lysine 1 mg/mL (PDL), which increased the adhesion between the PDMS and the collagen gel, and were incubated for 4 h at 37 °C.

**Figure 1:**
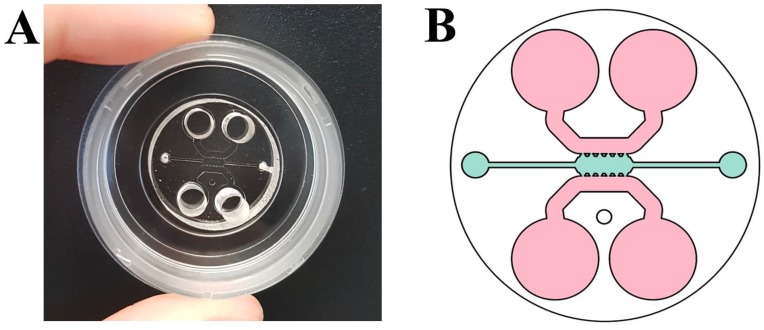
(**A**) Microfluidic device; and (**B**) scheme of the microfluidic device used in which the central channel where cells were cultured is shown in blue and the reservoirs are in pink [16].

The microfluidic device used consists of a central channel 1.3 mm wide and 2.5 mm long in which three-dimensional culture was carried out. In addition, it had two adjacent channels of 0.92 mm wide, which were used as reservoirs to add the culture medium necessary for cell survival (Figure 1A,B) [16]. Five PDMS posts had a trapezoid shape, which guaranteed the correct confinement of the gel and separated the central chamber and side channels.

### 2.3. Migration Assay

Both dermal fibroblasts (NHDF) and cardiac fibroblasts (NHCF-v), at a final concentration of 1 × 10^5^ cell/mL, were embedded in a collagen matrix type I with a final concentration of 4 mg/mL, with an optimal ratio of NaOH/H_2_O to obtain a physiological pH of 7.5. Collagen type I is the most abundant protein in the extracellular matrix in a human body [19], letting this scaffold to represent the physiological microenvironment. The gel was introduced into the central channel of the microfluidic device and polymerized at 37 °C in a wet chamber, turning the device every 5 min for at least 20 min. Collagen polymerization occured by an increase of temperature to room temperature at 37 °C, passing from a liquid state to another in gel form. Finally, the reservoir channels were filled with the appropriate medium and incubated overnight at 37 °C.

Having previously demonstrated the ability to generate chemical gradients in microfluidic devices [15,16], the culture medium was removed from one reservoir and filled with the appropriate treatment to create the gradient: Culture medium (control), culture medium with 5 ng/mL PDGF-BB [16], and culture medium with 15 ng/mL TGF-β1 [20,21]. The devices were analysed with phase contrast microscopy (Nikon D-Eclipse C1 Confocal Microscope, 10× lens, Nikon Instruments, Tokyo, Japan), taking images every 20 min for 24 h [14]. Results were analysed with a custin cell tracker algorithm designed in-house for use in MatLab (Mathworks, Natick, CA, USA) [22] and statistical analysis was performed with one-way anova test.

### 2.4. Immunofluorescence

To analyse differences in the morphology of cells, we decided to stain elements of the cytoskeleton, in particular, actin filaments and the vinculin, which affix actin to the membrane. In addition, nuclei were stained to show a more realistic image. For this staining, a concentration of 5 × 10^4^ cells/mL of dermal and cardiac fibroblasts was seeded in the central channel of the microfluidic device and the gel was polymerized, as explained above. After incubating overnight at 37 °C, the medium of the upper reservoir was removed and the culture medium (control), culture medium with 5 ng/mL PDGF-BB, or culture medium with 15 ng/mL TGF-β1 was added and devices were incubated overnight at 37 °C. The next morning, cells were fixed with 4% paraformaldehyde for 20 min and washed with phosphate-buffered saline (PBS). Subsequently, they were permeated with 0.1% Triton X-100 in PBS for 10 min at room temperature and washed again with PBS. Devices were blocked with 5% bovine serum albumin (BSA)/PBS for 4 h and incubated with a vinculin monoclonal antibody, Alexa Fluor 488, overnight at 4 °C. After several washes, cells were incubated with phalloidin-TRICT (1:100) and 4’,6-diamino-2-fenilindol (DAPI) (1:50) at 0.5% BSA/PBS for 3 h at room temperature in the dark and washed with PBS. Their maximum intensity projections were obtained from 3D images using confocal microscopy at 40× lens and cell shapes were analysed with a MatLab script developed in-house [23].

## 3. Results and Discussion

After epithelial and cardiac injury, healthy fibroblasts from nearby areas migrate to the damaged area to regenerate tissue [24,25]. During injury regeneration, different chemical factors are released to heal the tissue. In this work, a comparative study of the migration patterns of two types of fibroblasts, dermal (NHDF) and cardiac (NHCF-v) fibroblasts, was carried out under different controlled chemical gradients.

### 3.1. Cardiac Fibroblasts Migrate at Higher Speeds and a More Directional Pattern than Dermal Fibroblasts

**Figure 2:**
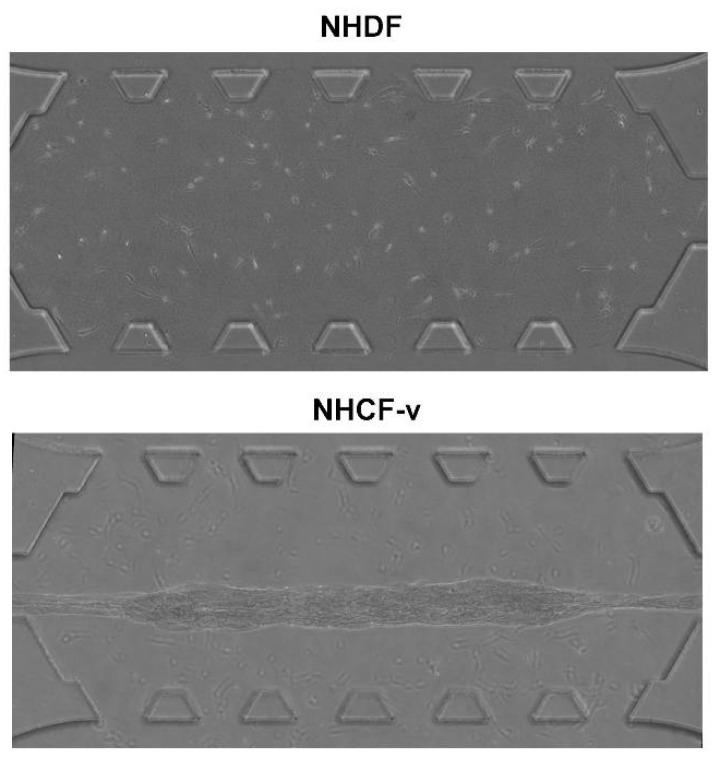
Dermal (NHDF) and cardiac (NHCF-v) fibroblasts seeded at a 3 × 10^5^ cells/mL concentration in a 2 mg/mL collagen gel, after 24 h of incubation. *n* = 3.

First, both cell types were seeded at the same density, 3 × 10^5^ cells/mL, into a collagen gel and incubated overnight at 37 °C to study their behaviour in 3D. Surprisingly, they showed a significant difference in contractile force. It was observed that cardiac fibroblasts were able to break the bonds between collagen and PDMS posts (Figure 2), while the gel that embedded the dermal fibroblasts remained fixed to the posts (Figure 2). This fact allows the conclusion that cardiac fibroblasts exerted higher forces than dermal fibroblasts.

**Figure 3:**
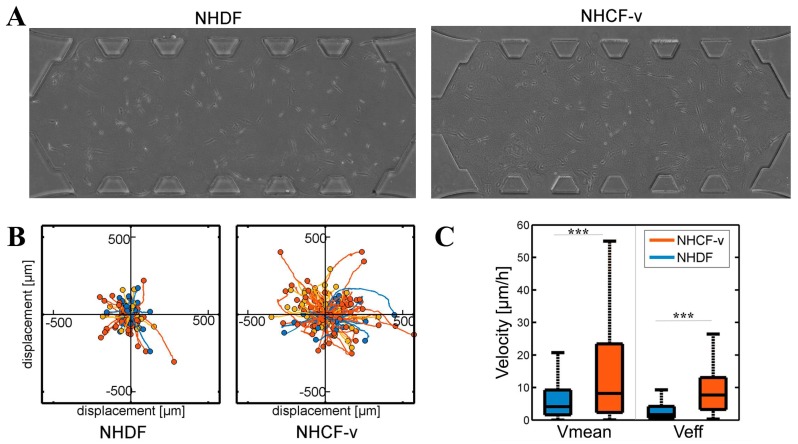
(**A**) Dermal (NHDF) and cardiac (NHCF-v) fibroblasts migration assay in 4 mg/mL collagen gels; (**B**) relative trajectories; and (**C**) mean and effective velocities. *** *p* < 0.005. *n* = 6. *n* = 6 devices and a mean of 25 cells per device.

Then, the basal migration of both cell types was analysed without any external stimuli in gels of 4 mg/mL, with three parameters measured: The distance travelled, the mean velocity, and the effective velocity, which is defined as the linear distance between the starting point and the final point divided by the invested time. Cardiac fibroblasts showed longer travelled distances (Figure 3B) and higher mean and effective velocities (Figure 3C) than dermal fibroblasts. In addition, due to the lack of external stimulus, both cell types showed a non-directional migration pattern (Figure 3B). 

These significant differences could be due to their cellular shape and their ability to exert force of each cellular type. Cardiac fibroblasts adopted longer shapes (Figure 3A) that might allow them to interact with more distant collagen fibres to push themselves in that direction, travelling longer distances (Figure 3B). In addition, due to the greater contractile force they showed (Figure 2), cardiac fibroblasts travelled at higher speeds, which resulted in a higher mean velocity (Figure 3C). Despite the greater dispersion shown by the mean velocity, the effective velocity was, practically, similar to the mean velocity (Figure 3C), indicating that, in cardiac fibroblasts, a directional advance movement predominates. On the other hand, the more rounded cellular shape of the dermal fibroblasts (Figure 3A) only allowed them to interact with nearby collagen fibres, thus, advancing shorter distances (Figure 3B) and, due to their lower contractile force (Figure 2), at a slower velocity (Figure 3C). Finally, dermal fibroblasts showed an effective velocity much lower than the mean velocity, indicating that a non-directional movement of little advance prevails (Figure 3C). For a better visual understanding, Supplementary Material contains a video of NHDF and NHCF-v migration in control conditions. However, it must be taken in account that media used for these cell types contained a different percentage of FBS, which could be affecting the migration pattern. Thus, as future work, we have the intention to repeat these assays culturing both types of cells with FGM-2 media, which contains 2% FBS.

### 3.2. Dermal Fibroblasts Are Chemoattracted by PDGF-BB, Whereas Cardiac Fibroblasts Respond to TGF-β1, Increasing Their Mean and Effective Velocities

The aim was, then, to determine the effect of different growth factors on fibroblast migration. Due to the importance of PDGF and TGF-β in wound healing and regeneration [5], they were considered of great interest for this study. Both of these factors present different isoforms, but it was decided to work with PDGF-BB, since it is involved in myocardial remodelling after heart attacks [6]. As for TGF-β, its three isoforms are present in wound healing, but a decrease of TGF-β1 is observed in non-pathological conditions, suggesting a greater participation of this isoform in cardiac regeneration [7].

**Figure 4:**
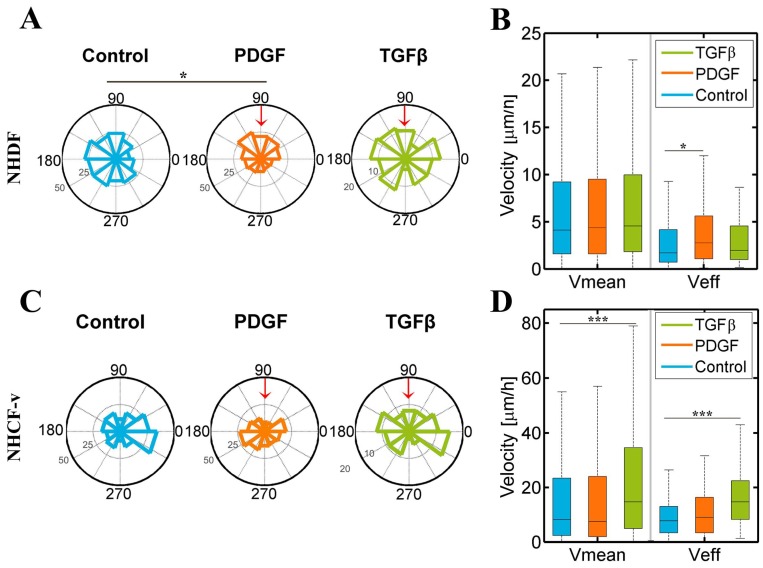
Dermal (NHDF) and cardiac (NHCF-v) fibroblasts’ migration assay in 4 mg/mL collagen gels stimulated with PDGF-BB (platelet derived growth factor-BB) and TGF-β1 (transforming growth factor beta 1) gradients, generated in the upper reservoir of the device that corresponds with 90° in the graphic and indicated by the red arrow: (**A**) Directional migration of NHDF, considering the length of the radio as the number of cells migrating in each direction; (**B**) mean and effective velocity of NHDF; (**C**) directional migration of NHCF-v, considering the length of the radio as the number of cells migrating in each direction; and (**D**) mean and effective velocity of NHCF-v. * *p* < 0.05, *** *p* < 0.005. *n* = 6 devices and a mean of 25 cells per device.

It was observed that dermal fibroblasts’ migration pattern had a significantly different distribution in response to PDGF-BB when compared to control conditions, tending to migrate towards PDGF-BB, a factor that also increases their effective velocity. On the other hand, cardiac fibroblasts only responded to TGF-β1, increasing their mean and effective velocity (Figure 4). 

Previous studies have shown that, in wound conditions, dermal fibroblasts are chemoattracted by PDGF-BB, both in 2D [26] and 3D [16]. Our results might reaffirm this fact, showing a low tendency for NHDF to migrate in the direction of the growth factor gradient (Figure 4A). However, not only may they be attracted, but this factor can increase their effective velocity (Figure 4B), which suggests that a change in the cytoskeleton of fibroblasts might provoke a modification of their type of motion, inducing a more directional movement. On the other hand, cardiac fibroblasts were not attracted by any of the factors (Figure 4C), but there was a very significant increase in their mean and effective velocity in response to TGF-β1 (Figure 4D).

### 3.3. PDGF-BB and TGF-β1 Do Not Modify Dermal or Cardiac Fibroblast Morphology

**Figure 5:**
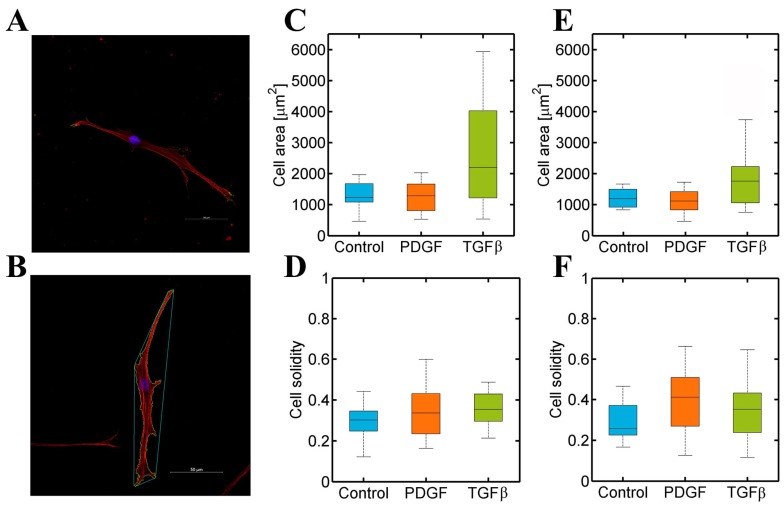
(**A**) Normal Human Dermal Fibroblast cultured in a 4 mg/mL collagen gel were stained for actin (red), vinculin (green), and nucleus (blue). Images were captured with a confocal microscope; (**B**) an example of an NHDF in control conditions analysed with the developed MatLab script. Cells were stained for actin (red) and nuclei (blue), and images were acquired with a confocal microscope. (**C**–**F**) Dermal (NHDF) and cardiac (NHCF-v) fibroblasts’ morphology analysis in 4 mg/mL collagen gels stimulated with PDGF-BB and TGF-β1 gradients: (**C**) Cell area of NHDF; (**D**) cell solidity of NHDF; (**E**) cell area of NHCF-v; and (**F**) cell solidity of NHCF-v. *n* = 10–15.

To determine if cell motility changes, in response to growth factors, are related to a cellular shape modification, fibroblasts were stained with phalloidin and DAPI to visualise their actin, vinculin, and nuclei (Figure 5A). Then, their cell area and solidity were analysed. The former was calculated by segmentation of the cells from the image. The latter was calculated by dividing the area of a cell by the area of the minimum convex polygon containing the cell (Figure 5B). Thus, solidity data showed the branched shape of cells. The smaller the values were, the longer the protrusions, and the thinner the body of the cells were. 

Figure 5 showed a similar cell area in dermal and cardiac fibroblasts (Figure 5C,E) and, also, their cell solidity was approximately similar (Figure 5D,F), minor to 0.5, indicating both cell type shapes were significantly branched. However, Figure 5 showed no change in both types of fibroblasts under different conditions. Thus, PDGF-BB and TGF-β1 altered migration patterns in dermal and cardiac fibroblasts without altering their morphology, which might imply an intracellular regulation.

## 4. Conclusions

Fibroblast migration to damaged areas is essential to achieve a successful wound healing process and, as was shown in this work, affected tissue and associated cells are relevant in the process. Dermal fibroblasts had a different behaviour response to wound growth factors than that of cardiac fibroblasts. Thus, it is logical that wound healing in the epidermis and heart is different. This study demonstrates the versatility of microfluidic devices for evaluating the impact of chemical gradients on 3D fibroblast migration, which can be used to recreate numerous physiological environments and evaluate the effect of a large variability of molecules.

## Supplementary Materials

The following are available online at http://www.mdpi.com/2306-5354/5/2/45/s1.

## Nomenclature

2D	two dimensions
3D	three dimensions
BSA	bovine serum albumin
DAPI	4′,6-diamino-2-fenilindol
FBS	fetal bovine serum
GA-1000	gentaminicin sulfate/Amphotericin
NHCF-v	normal human cardio fibroblasts from ventricles
NHDF	normal human dermal fibroblasts
PBS	phosphate-buffered saline
PDGF-BB	platelet derived growth factor BB
PDL	poly-d-lysine
PDMS	polydimethylsiloxane
rhFGF-B	Fibroblast Growth Factor basic human recombinant
TGF-β1	transforming growth factor beta 1
